# Use of Angong Niuhuang in Treating Central Nervous System Diseases and Related Research

**DOI:** 10.1155/2014/346918

**Published:** 2014-12-18

**Authors:** Yu Guo, Shaohua Yan, Lipeng Xu, Gexin Zhu, Xiaotong Yu, Xiaolin Tong

**Affiliations:** ^1^Endocrinology Department of Guang An Men Hospital, China Academy of Chinese Medical Science, Beijing 100053, China; ^2^Beijing University of Chinese Medicine, Beijing 100029, China

## Abstract

In Chinese medicine-based therapeutics, Angong Niuhuang pill (ANP) is one of the three most effective formulas for febrile diseases, and it is also used to treat other diseases. This paper reviews current knowledge regarding the clinical and pharmacological effects of ANP for treating different central nervous system (CNS) diseases to confirm its validity and efficacy. These diseases are like centric fever, coma, stroke, and viral encephalitis. This review reveals that various diseases could be treated using the same agent, which is one of the most important principles of traditional Chinese medicine (TCM). According to the “*Same Treatment for Different Diseases*” principle, ANP might be efficacious in other CNS diseases.

## 1. Introduction

The premier description of Angong Niuhuang pill (ANP) is in* Shang Jiao*, Volume One of the* Treatise on Differentiation and Treatment of Epidemic Febrile Diseases*, written by* Wu Jutong* in the Qing Dynasty.

CNS diseases manifest mainly at the neural tube, neural crest, spinal cord, and brain. Typical clinical manifestations include high fever, headache, dizziness, unconsciousness, nausea and vomiting, difficulty moving, hemiplegic paralysis, language disorders, weakness, neck rigidity, and epilepsy. In ancient times ANP was known as the pill that could rescue the patient immediately and help revive those who were on the brink of death. It was named “*one of the three treasures,*” and was used to treat high fevers [1]. Today, ANP has the potential to provide new breakthroughs for the treatment of CNS diseases such as stroke, coma, centric fever, and viral encephalitis, as well as the design of clinical studies of these diseases.

## 2. Clinical Applications

### 2.1. Stroke

Strokes can be categorized as ischemic or hemorrhagic. Cerebral hemorrhage is a brain parenchymal nontraumatic hematoma that is primarily caused by hypertension and the subsequent clogging of arteries. It normally causes intracerebral hematoma; however, the blood sometimes penetrates the brain parenchyma, which results in intraventricular and subarachnoid hemorrhage. The clinical features of this type of stroke are dizziness, headache, vomiting, loss of consciousness, hemiplegia, hemidysesthesia, and hemianopsia [2]. On the contrast, hypoxic-ischemic encephalopathy is caused by circulatory or respiratory diseases, which results in an insufficient oxygen supply to the brain. If the brain arterial oxygen partial pressure falls below 3132 kPa, a lack of oxygen diffusion will result in collateral damage to the brain [3].

However, in the theoretical system of traditional Chinese medicine, stroke that results from impaired function of the* liver*,* kidney*,* heart*,* spleen*, and other viscera produces a series of pathological changes including* wind* (mainly in* liver*),* internal heat* (*liver* and* heart heat*),* phlegm* (*wind* and* wet phlegm*),* qi* (*deficiency*,* reverse flow*, and* stagnation*), and* blood* (*deficiency* and* stasis*). Stroke disturbs* qi*-*movement* and leads to a* qi-blood imbalance*, which are all the results of* external wind*, emotional changes, improper diet, too much physical and mental labor, or excessive sexual activities [4]. She and Gao [5] ascribed the basic theory of ANP to* wind-yang excess*. They found that* wind phlegm* and* phlegm fire* disturbed the upper body, which aroused mental confusion. The key steps of treatment can bring down a fever, detoxifying and resolving turbidity and inducing resuscitation.

Zhan [6] randomized 368 intraventricular hemorrhage patients into an observation group and a control (C) group. In addition to conventional treatment, the observation group was also given with Xing Nao Jing injections, ANP, and acupuncture. The results showed that intracerebral pressure, temperature, and the time to recover consciousness were significantly better in the observation group than in the C group (*P* < 0.05). After 10 days of treatment, the nerve function defect grade and the scores of TCM syndrome were all lower in the observation group than in the C group (*P* < 0.01).

Moreover, Zhang and Li [7] divided 180 cerebral hemorrhage cases in average into two groups, which were treated with biomedicine or a combination of biomedicine and Angong and Qingkailing injections. The results revealed an increased curative effect and treatment efficacy in the combination group compared with the biomedicine group (*P* < 0.05). The level of disability and death rate in the combination group were also lower than in the biomedicine group (*P* < 0.05). These findings suggest that combining TCM and biomedicine is effective for reducing the mortality rates after cerebral hemorrhage.

In addition, Xing et al. [8] randomized 54 patients with cerebral paralysis into a treatment (T) group, which was given with Angong dissolved in warm water using a nasogastric tube as well as regular emergency treatment, and a C group, which received cytidine diphosphate choline, tranquilizers, and glucocorticoids plus cooling. T group exhibited a cooled fever (*P* < 0.01), coma (*P* < 0.05), and convulsions compared with C group.

Wu et al. [9] confirmed the benefits of combining ANP with biomedicine to treat damaged nerve function in patients with acute cerebral infarction. After treatment, plasma brain natriuretic peptide (BNP) and C-reactive protein (CRP) levels were decreased more markedly in the combination treatment group compared with the C group (*P* < 0.05). These authors stated that use of ANP to reduce plasma BNP and CRP levels positively affected the neural function of patients with acute cerebral infarction.

Research regarding the clinical efficacy and safety of ANP as an adjuvant treatment for moderate or severe neonatal hypoxic-ischemic encephalopathy (NHIE) revealed that it affects NHIE. Specifically, it could promote patients recovery and decrease the occurrence of complications [10]. Huang et al. [11] reported the clinical effects of ANP during the treatment of hypoxic-ischemic encephalopathy. Sixty patients with hypoxic-ischemic encephalopathy were randomized into a C group given conventional Western medical treatment and an observation group that also received ANP. Conscious recovery time, temperature, the occurrence of seizures, the outcome of the Glasgow Coma Scale (GCS), and clinical efficacy were compared between groups. The observation group had higher GCS scores (*P* < 0.02) than the C group. The observation group also had more stable body temperatures and showed fewer convulsions compared with the C group. All comparisons were statistically significant (*P* < 0.05). Xiao [12] randomized 60 patients with ischemic stroke and a diagnosis of phlegm-heat syndrome into either a comparison group, which received Western medical treatment, or an observation group that was administered the basic Western treatment as well as ANP (one pill orally twice a day). The TCM syndrome scores that evaluated routine daily living activities, assessed changes in neurological function, and analyzed the standards of clinical efficacy revealed that the basic characteristics of the two groups were not significantly different. Following treatment, the TCM syndrome scores of the observation group regarding neurological function, daily activities, and overall efficiency were 80%, 83.3%, and 83.3%, respectively, compared with 60%, 43.3%, and 60% in the comparison group. The results revealed that ANP was highly effective for curing ischemic stroke and phlegm-heat syndrome.

### 2.2. Coma

During a coma the depression of cortical and subcortical mesh structures and functions results in severely disturbed consciousness. Patients might become semiconscious or unconscious, which is apparently a lack of reaction to pain stimuli and of voluntary movement. Because of the suppression of higher nerve activity, various external stimuli are unable to trigger actions including motor reflexes, swallowing, and reaction to light [13]. There are two etiologies of coma. One results from a primary injury to the cerebral cortex, diencephalon, midbrain, and the top of the pons, whereas the other might result from brain injury, secondary systemic toxicity, metabolic blocks, or an endocrine imbalance [14].

#### 2.2.1. Coma Caused by Brain Trauma

Skull damage results in intracranial hemorrhage, intracranial hematoma, increased intracranial pressure, ischemia caused by decreased cerebral blood flow, hypoxia, cerebral edema, the inflammatory response, and apoptosis, which together give rise to brain damage and coma. A delay in treating a skull damage-induced coma might lead to the formation of a cerebral hernia, which has the highest mortality and disability rate among all bodily injuries [2]. Chen and Wang [15] randomized comatose traumatic brain injury patients to either treatment (*n* = 133) or control (*n* = 1790) groups. Patients in both groups received the same standard care, but patients in T group were also given ANP twice daily (3 g each) for a week. The outcomes of Glasgow Coma Scale (GCS) and Acute Physiology and Chronic Health Evaluation II (APACHE II) showed that T group had significantly lower level of high-sensitivity CRP and less severe epilepsy. Therefore, it was concluded that ANP had a significant effect on the treatment of comatose traumatic brain injury patients. Wang [16] divided 100 comatose traumatic brain injury patients with GCS scores <8 into treatment and control groups of 50 patients. Both groups received treatment to prevent dehydration, maintain hemostasis, reduce intracranial pressure, and prevent infection. Patients in the treatment group were given additional treatments including ANP and head electroacupuncture combined with hyperbaric oxygen therapy. After 2 weeks the T group had a better sober rate (*P* < 0.001) and GCS scores (*P* = 0.002) than the control group. Furthermore, the T group had significantly lower rates of gastrointestinal bleeding (*P* = 0.005) and pulmonary infection (*P* = 0.015) than the control group, which suggests that the addition of ANP and head electroacupuncture plus hyperbaric oxygen therapy to the normal treatment increased the therapeutic effectiveness and decreased the rate of complications.

Lin et al. [17] treated comatose traumatic brain injury patients by using naloxone only in the control group and naloxone together with ANP in the treatment group. They reported significant differences in sobriety and intense coma (mortal) rates between the two groups (*P* < 0.05). There was also a big difference in GCS between the treatment (35.6 ± 1.8) and control (19.3 ± 1.2, *P* < 0.01) groups. Data revealed that the combined use of ANP and naloxone was more effective for the treatment of comatose traumatic brain injury patients than use of naloxone alone. Wang et al. [18] evaluated the effect of combining ANP with conventional therapy on brain trauma-induced diffuse axonal injury. Compared with the control group that received conventional therapy alone, the combined therapy improved consciousness disorder, body temperature, and decerebrate tetanus in patients to yield favorable prognosis. Li et al. [19] found that the recovery time and disease course in patients treated using ANP combined with biomedicine were shorter than in patients only treated with biomedicine. In a prospective randomized clinical trial of 104 craniopathy patients with unconsciousness, the addition of ANP to routine treatment significantly increased the efficiency (*P* < 0.005) of processes used to enhance the excitation processes within the cerebral cortex of unconsciousness patients [20].

#### 2.2.2. Coma Caused by Blocked Cerebral Blood Circulation

Li et al. [21] assessed the effects of ANP on severe cerebral vascular disease in elderly individuals. The research team divided 50 patients into a control group that received regular treatment and the second group that were administered regular treatment plus ANP. The total rate of effectiveness, which was dependent on the clinical neural function, was higher in the treatment group (76.92%) than in the control group (58.33%, *P* < 0.05). The GCS scores were increased significantly from baseline in both groups (*P* < 0.05), but the score in the treatment group was higher than that in the control group (*P* < 0.05). In addition, the reduced neurological deficit scores were higher in the treatment group than in the control group. Statistics revealed that ANP could promote the recovery of awareness in patients with severe cerebral vascular diseases. Shi [22] evaluated the effect of the combination of TCM and biomedicine in 28 patients with coma caused by brainstem hemorrhage, which shows the benefits of combining Chinese and biomedicine are obvious. Zhang and Qu [23] learnt the effect of ANP combined with biomedicine for treating hypertensive cerebral hemorrhage. The time of coma in the treatment group (ANP plus biomedicine) was shorter than that in the control group (biomedicine only; *P* < 0.05). Consistent with this, the neural disorder scores were lower in the treatment group than in the control group (*P* < 0.05). However, there were no significant differences in blood pressure fluctuation or mortality rate between the two groups. Therefore, ANP promoted recovery from coma and improved patient prognosis. Qiu and Wu [24] studied 60 first-episode patients with an altered mental status following acute cerebral infarction. All patients received conventional treatment, but 30 patients were also administered ANP nasally. The GCS scores of all 60 patients improved greatly. However, there were statistically significant differences in the improvement between the therapy and control groups regarding hematocrit, fibrinogen, and blood viscosity (*P* < 0.01). Therefore, ANP significantly inhibited platelet aggregation, prevented blood clots, and lowered blood viscosity to facilitate the reversal of narcosis.

#### 2.2.3. Infantile Coma

Li treated 30 children with infantile coma by using ANP combined with biomedicine [25]. After the treatment all but two of the children became fully conscious. These results suggest that ANP plus biomedicine has a huge curative effect on infantile coma and that ANP could counteract adverse reactions to sedative drugs such as diazepam and phenobarbital sodium.

#### 2.2.4. Coma Caused by Infectious Diseases

Wang and Zhang [26] evaluated the effect of combining ANP with biomedicine on infectious disease-induced coma. Based on TCM syndrome differentiation, patients in the treatment group were given different decoctions in addition to ANP. Patients in the treatment group had significantly higher GCS scores than the control group (*P* < 0.05), which confirms the applicability of the ANP toward promoting the heat-clearing and detoxifying functions, brain activity, and regaining consciousness.

### 2.3. Centric Fever

Centric fever is a nonpyrogenic fever resulting from centric damage and an increased temperature in the centrum. The main clinical feature is a constant fever that is caused by the effects of cerebrovascular disease, cranial trauma, and Malin syndrome [27]. Shi and Tang [28] explored the antipyretic effects of ANP on centric high fever in 37 patients with different TCM syndromes including* qi* system* heat excess syndrome* in the channels and collaterals (20 cases) and* heat excess syndrome* and* fu organ constipation* in the internal organs (17 cases). The data revealed that ANP exerted definite antipyretic effects on a centric high fever because the 100% effectiveness rate was observed. Zhang et al. [29] randomized 206 cranial trauma patients into groups that received ANP combined with biomedicine (CW; *n* = 126) or biomedicine only (W; *n* = 80). ANP was administered nasally by tube or orally depending on the TCM syndrome. The CW group had a significantly greater improvement than the W group (*P* < 0.01). In an additional study, Feng [30] randomly assigned confirmed centric fever patients into treatment (T; *n* = 33) and comparison (C; *n* = 32) groups, and using basic physical cooling methods treated both groups, and the T group also received ANP (orally/lavage/enema). The T group had an effectiveness rate of 93.94%, which was significantly higher than the C group (78.13%; *P* < 0.01). Li and Bao [31] assigned 100 patients with cranial trauma, centric fever, and similar baseline characteristics into groups that received a regular Western treatment for centric fever (group C; *n* = 50) or Western treatment plus ANP (group T; *n* = 50). The T group had a mean cooling time of 23 h, compared with 48 h in the C group. A follow-up survey revealed that the T group survivors had a better quality of life. Zhang and Wang [32] assessed the effect of ANP in patients with centric fever that was caused by cerebral hemorrhage. The overall effectiveness of treating centric fever by using traditional and biomedicine (treatment group; *n* = 36) was higher than using biomedicine alone (control group, *n* = 36; *P* < 0.01). Jiang [33] combined ANP via nasal feeding with biomedicine to treat 18 patients with fever that was caused by massive cerebral infarction and treated without mild hypothermia and hibernation therapy. The results showed that the effective rate of cooling and recovered consciousness was 88.9%. Therefore, ANP is highly effective at increasing the recovery of consciousness and cooling body temperature in patients with massive cerebral infarction and a high fever.

### 2.4. Viral Encephalitis

Viral encephalitis is an acute intracranial inflammation that is caused by a variety of viruses and is characterized by fever, headache, vomiting, unconsciousness, or mental abnormalities. In mild cases full recovery is possible without medical treatment, whereas severe cases can result in death or complications. Meningitis is caused by several different viruses and has different seasonal incidence and typical clinical features.

In terms of TCM, viral encephalitis is caused by exposure to exogenous pathogenic heat, including* wind heat*,* summer heat*, and* dryness heat*. The* lung* and* stomach* are the foci of the lesions involving the* heart* and* liver*. During the recovery stage the lesion organs are the* spleen*,* liver*, and* kidneys*. The disease results from the conversion of exogenous pathogenic* heat* into* dryness* after the* heat-fire* attack to the human body. Viral encephalitis is a common acute disease with a high prevalence and complicated course; the extreme heat causes* wind* and* fire*. The pathogenic condition spreads and changes according to the system of the Defense-*qi*-Nutrient-Blood, as well as* heat*,* phlegm*, and* wind* [34].

Yu [35] randomly assigned 30 children with viral encephalitis into treatment (T; *n* = 13) and comparison (C; *n* = 17) groups. Both groups were given conventional Western treatments such as antiviral agents, treatment for dehydration, procedures to decrease the intracranial pressure, and anticonvulsants. The T group was also given ANP via the mouth or nasal feeding. The clinical evaluation and clinical course were better in the T compared with the C group (*P* < 0.05). A retrospective clinical analysis by Yao et al. [36] was performed on the clinical data of 92 patients with viral encephalitis. The recovery rate and CT or MRI images indicated that the outcome of the group treated with ANP, acyclovir, mannitol, and adrenocortical hormone was better than that of the biomedicine group.

Wang and Dong [37] measured cerebrospinal fluid NO and TNF-*α* levels in three children groups suffering from viral encephalitis, febrile seizures, and ordinary trauma, respectively. The concentrations were higher in the viral encephalitis group than in the febrile seizures group and ordinary trauma group, which shows NO and TNF-*α* participate in inflammation of the CNS. Zhang et al. [38] evaluated the treatment of 97 children with viral encephalitis using oral or nasal ANP combined with basic Western treatment. The total rate of effectiveness was significantly higher (92.9%) in the group given ANP than in the comparison group (73.4%; *P* < 0.05). The levels of TNF-*α* in the cerebrospinal fluid, a standard marker used to assess the effectiveness of treatment, declined sharply in both groups. Therefore, ANP could decrease TNF-*α* levels, improve recovery rates, and reduce the treatment periods. Liang [39] reported the clinical effect of combining Chinese and biomedicinal therapy to treat viral encephalitis in children. TNF-*α* levels in the treatment group, which were treated using ANP plus biomedicine, declined more steeply than did those in the control group, which were treated using only Western medicine (*P* < 0.05). Therefore, the data reveal significant advantages of combining Chinese and biomedicine to treat viral encephalitis.

**Figure 1:**
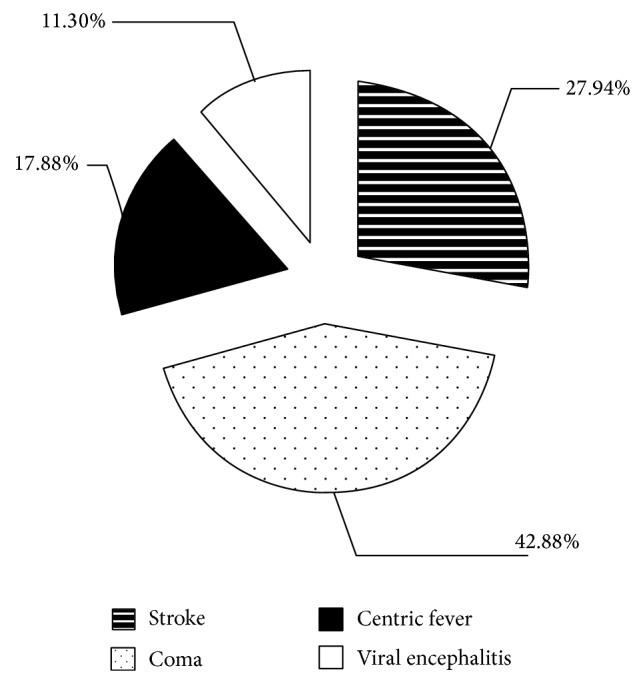
The distribution of severe nerve disease cases treated with ANP.

A total of 2548 cases of severe nerve disease that were treated using ANP were analyzed in the clinical studies described above. Among these, 1108 coma cases comprised 42.88% of the total cases, which was the highest percentage of the conditions. Analysis of the distribution of the severe nerve disease cases revealed that coma and viral encephalitis were the most and least prevalent, respectively (Figure 1).

## 3. Pharmacological Studies

### 3.1. Cerebral Protection

#### 3.1.1. Reducing Brain Edema

Cephaledema often follows intracerebral hemorrhage. Its causes are complex and lead to pathological changes in physiology and function such as the formation of intracerebral hematomas, decreased cerebral blood flow, ischemic anoxia of brain tissue, and metabolic disorders [32]. Matrix metalloproteinases, particularly matrix metalloproteinase-9 (MMP-9), are closely related to the formation of cephaledema and destruction of the blood-brain barrier because they can degrade all components of the extracellular matrix [40]. Jiang et al. [41] found that changes in serum MMP-9 levels were correlated with the evolution and volume of brain edema, and that they had a direct effect on the condition. ANP can reduce brain edema after cerebral hemorrhage by regulating the expression of MMP-9. Yin et al. [42, 43] performed two animal experiments to demonstrate that ANP can modulate the expression of MMP-9 and effectively reduce brain edema in rats with experimental cerebral hemorrhage. Zhu [44] used the Feeney assay in a rat model of closed brain injury to demonstrate that the administration of ANP resulted in a greater decrease in brain water content and cerebral cortex Evans Blue dye content compared with the model control and Nimotop treatment groups. In the ANP-added group, an increase in synaptic density in the lateral ventricle was observable using electron microscopy. Therefore, ANP could reduce brain edema by decreasing capillary permeability and improving the tolerance to ischemia or hypoxia, thereby protecting the organization of the brain. In a rat model of middle cerebral arterial occlusion, Zhao [45] found that ANP, both with and without the coadministration of cinnabar and realgar, could dramatically reduce the cerebral infarction area and brain water content, increase the levels of catalase (CAT) and glutathione peroxidase (GPX), and lower the levels of lipid peroxide (LPO) and lactic acid (LD). This suggests that ANP could protect the animals from cerebral ischemia in a way that might be related to its antioxidant effects.

#### 3.1.2. Anti-Inflammatory Effects

Yin [46] developed a rat intracerebral hemorrhage (ICH) model by injecting anticoagulated arterial blood into the caudate nucleus. The inflammatory cells around the hematomas were then counted using H&E staining. In addition, RT-PCR was used to assess the expression of TNF-*α* mRNA, and an ELISA was used to measure TNF-*α* protein. ANP reduced the number of inflammatory cells around the hematomas, inhibited the expression of *TNF-α* mRNA and protein, and inhibited the inflammatory reaction in rats with ICH.

#### 3.1.3. Neuroprotective Effects

Zhang et al. [47] used primary rat midbrain neuronal-glia cultures as an* in vitro* model to measure the neuroprotective effects of different agents. Compared with the normal control group, the number of dopamine (DA) neurons in the lipopolysaccharide (LPS) model group was decreased by 40% (*P* < 0.05). LPS also activated the microglia. The expression of TNF-*α* and* iNOS* mRNA was increased in the microglia in LPS control group (*P* < 0.05). Compared with the LPS control group, LPS-mediated DA neuronal loss was attenuated significantly by 40% and 30% by the administration of ANP and realgar, respectively (*P* < 0.05), whereas the activation of microglia and the expression of *TNF-α* mRNA were decreased by 61% and 52% (*P* < 0.05). These results demonstrate that ANP protects against LPS-induced neurotoxicity via anti-inflammatory properties and suggest that realgar might play a key role in the neuroprotective effects of ANP. In contrast, cinnabar did not exert any neuroprotective effects. Li et al. [48] studied heat shock protein 70 (HSP70) expression in rats with cerebral ischemia injury. The infarct volume was significantly smaller in the ANP group compared with that in the ischemia group (*P* < 0.05). HSP70 expression was significantly higher in the ANP group than in the ischemia group. ANP increased HSP70 expression and protected brain cells from cerebral ischemia beginning on day 3.

Zhong et al. [49] found that ANP could promote angiogenesis in the cerebral cortex and striatum of ischemic rats and also increase the amount of blood vessels in the injured area. It also increased neurogenesis in the subgranular zone (SGZ), subventricular zone (SVZ), cortex, and striatum. Experimental results assessing the protective effects of ANP in a model of experimental cerebral ischemia in rats showed that it could not only decrease the cerebral water content, but also increase the serum levels of interleukin-10 (IL-10) [50]. This inhibits the expression of the inflammatory cell factors IL-1, IL-6, and IL-2 and their receptors, thereby reducing inflammatory reactions and tissue injury [51]. During cerebral ischemia and cerebral infarction, IL-10 facilitates recovery and the survival of injured neurons by alleviating inflammation and inhibiting neuronal apoptosis in areas of cerebral hemorrhage [52, 53]. A recent review discussed the role of ANP in inhibiting nerve cell apoptosis by upregulating the phosphorylation of Akt, which is the key molecule for the survival of motor neurons following cerebral ischemia [54].

#### 3.1.4. Effects on Nitric Oxide (NO) and Nitric Oxide Synthase (NOS)

NOS participates in the release of free oxygen radicals during brain ischemia-reperfusion. eNOS in vascular endothelial cells catalyzes the release of NO via L-arginine. In turn, NO maintains normal vascular function [55]. Xing and Zhang [56] randomized 68 patients with cerebral hemorrhage into groups that received cytidine diphosphate choline (observation group; *n* = 33) or ANP (comparison group, *n* = 35). One week later serum asymmetric dimethylarginine (ADMA) levels were reduced significantly in the observation group compared with those in the comparison group (*P* < 0.01). However, there was a significant increase in NO in the serum of the observation group (*P* < 0.01). Liu et al. [57] used a hypertensive (hypervolemic) rat model to demonstrate that the serum NO content was lower in animals treated using integrated Chinese and biomedicine compared with the other three groups (TCM group, biomedicine group, and control group) (*P* < 0.05). Data revealed that ANP combined with biomedicine could alleviate acute brain tissue injury during the acute stage of spontaneous hypertensive cerebral hemorrhage in rats. Yang et al. [58] verified the protective effects of ANP in acute cerebral hemorrhage by demonstrating that it could decrease NO levels, NOS activity, and the cerebral monoamine neurotransmitter content.

### 3.2. Resuscitation and Sedation Effects

When studying the sedative effects of ANP and its simplified prescription, Ye et al. [59] found that the administration of 0.75, 1.5, and 3.0 g kg^−1^ ANP once daily with 38 mg kg^−1^ pentobarbital sodium (below the hypnotic threshold) had more pronounced synergistic sedation effects than did the model control group (*P* < 0.005).

### 3.3. Antipyretic Effects

Experiments performed by Ye confirmed that ANP and its simplified prescription (without Zhusha and Xionghuang) exerted clear antipyretic and sedative effects in models including rabbits with high fevers caused by typhoid vaccine, sleeping rats caused by pentobarbital sodium, oxygen-deficient rats killed using NaNO_2_, and rats with eclampsia was caused by strychnine nitrate and PTZ [59]. Jin and Pan [60] administered 1.0 g/kg ANP by intraperitoneal injection to rabbits, and the rise in body temperature of the rabbits caused by the triple vaccine was clearly lower than the control group after 0.5–4.0 h.

With the development of pharmacological research, the main drugs in Angong Niuhuang prescriptions and their pharmacological effects have been identified, which revealed that Angong Niuhuang is effective for the treatment of diseases of the nervous system (Table 1).

## 4. STDDP Applied in CNS Diseases

Regardless of the cause and pathogenesis of the four CNS diseases described above, their symptoms overlap. For example, stroke patients and children with viral encephalitis can also be in a coma or have a high fever. Therefore, Chinese clinicians consider these different diseases from a TCM perspective and find the common features that represent the same TCM pathogenesis and then make a decision to use the same medicine or treatment according to the “*Same Treatment for Different Diseases*” principle (STDDP), which is a crucial principle of TCM.

STDDP has been widely applied in TCM. In addition to the diseases described above, ANP can also be used to treat pulmonary cerebral disease, insolation, and eclampsia, which can have common symptoms such as coma and high fever [61–63]. As a result, the use of ANP for centuries has suggested that it could be used to treat CNS diseases by both Chinese and Western clinicians.

## 5. Conclusion

Although the mechanism by which ANP cures these diseases and the active ingredients within ANP are yet to be elucidated, Chinese clinicians have used ANP to treat CNS diseases for hundreds of years, which suggested that ANP is both effective and safe.

We realize that clinical research is not an end but a way to solve clinical problems. With the development of evidence-based medicine, clinical research has developed from the case level to the population level, and best evidence has become the main principle of prescriptions. Findings have been reported and scrutinized, and TCM research should continue to be performed in real-world studies to ensure the reliability of the results.

The results of the literature are persuasive; however, there are some problems with the way the clinical conditions have been analyzed. (1) The clinical observations and research are of poor quality. For example, most of the studies on stroke patients, including cases of cerebral hemorrhage and cerebral ischemia, do not report the symptoms fully. Even the “*blockage syndrome*” and “*prostration syndrome*” deviate from the principle of combining disease, syndrome, and symptom. This could be attributable to a lack of response to treatment. (2) Few trials have studied the mechanism of central heating, which could be attributed to a lack of mature animal models. (3) There is small number of single case reports, which is consistent with the development of evidence-based medicine.

## Figures and Tables

**Table 1:** Main pharmacological ingredients and the pharmacological effects of the main constituent herbs.

Herbs [64]	Main pharmacological ingredients [65]	Pharmacological effects [65]
Bezoar	Bilirubin Bile acid Deoxycholic acid Taurine Cholesterol	Sedation Anticonvulsion Antipyretic Anti-inflammatory Antiviral Cardiotonic Antiplatelet Aggregation

Rhinoceros horn	Keratin Cholesterol Calcium phosphate Calcium carbonate	Sedation Anticonvulsion Antipyretic Anti-inflammatory Antiviral Cardiotonic Antiplatelet Aggregation

Radix scutellariae	Baicalin Baicalein Wogonoside Wogonin Neobaicalein	Anti-pathogeny microorganism Anti-inflammatory Antiallergy Antipyretic Sedation Hepatoprotective Cholagogue

Coptidis rhizome	Berberine	Anti-pathogeny microorganism Antipyretic Anti-inflammatory Antiallergy Cholagogue Sedation Hepatoprotective

Cape jasmine	Gardenoside Geniposide Genipin	Anti-pathogeny microorganism Anti-inflammatory Anti-allergy Antipyretic Sedation Hepatoprotective Cholagogue

Musk	Muscone	Double-acting role in regulating the CNS, anti-inflammatory Antiplatelet aggregation Cardiotonic

Borneol	Borneol	Double-acting role in regulating the CNS, anti-inflammatory Postoperative analgesia Antimicrobial Antimyocardial ischemia Antifertility

Curcuma	Curcumene Curcumine Turmerone Ar-turmerone	Anticancer Hepatoprotective Cholagogue Exciting gastrointestinal smooth muscle Immunosuppressive

## Research Highlights

(1) Because of its fever-reducing, detoxification, and decongestive actions, ANP has long been used to treat conditions such as coma, fever, and cephaledema in China.
(2) Modern pharmacological studies reveal that ANP affects the central nervous system by relieving cerebral edema, exerting antipyretic and anti-inflammatory effects, and allowing resuscitation and sedation, as well as other related actions.
(3) The indication for ANP in treating CNS diseases highlights the “*Same Treatment for Different Diseases*” principle.
